# In vitro gas production and rumen fermentation for diets containing increasing levels of *Panicum maximum cv. Mombasa* with or without spirulina

**DOI:** 10.1007/s11250-024-04262-x

**Published:** 2025-01-15

**Authors:** M. I. Meteab, M. M. Khorshed, Abeer M. EL-Essawy, M. S. Nassar, N. E. El-Bordeny

**Affiliations:** 1 Animal and Poultry Nutrition Department, Animal, and Poultry Division, Desert Research Center, Mataryia, Cairo Egypt; 2 Animal Production Department, Faculty of Agriculture, Ain-Shams University, 68 Hadayek Shoubra, Cairo, 111241 Egypt

**Keywords:** *Panicum maximum cv. Mombasa*, Alfalfa, Spirulina, In vitro, Gas production, Rumen fermentation

## Abstract

This study was designed to evaluate the effect of substituting alfalfa hay with graded levels *panicum maximum* without or with graded levels of spirulina supplementation on rumen fermentation and nutrient degradability. The evaluation was achieved through an in vitro study, rumen fluid was obtained from adult sheep aged 2 years (fed clover hay), immediately after slaughter. Experimental diets were formulated as isonitrogenous and isocaloric and contained 40% forage. Forage composition was altered by substituting alfalfa hay with graded levels of *panicum maximum* hay, so that treatment diets contained 0, 25, 50, 75, and 100% of the forage from *panicum maximum* for treatments R1, R2, R3, R4, and R5, respectively. Additionally, each treatment was further supplemented with graded levels of spirulina at the following rates: 0, 0.5, 1.5, 2, 2.5, and 3 mg/g. Results indicated that gas production after 24 h per g DM, OM and DDM has a positive linear relationship with spirulina supplementation level (R^2^ = 0.80, 0.83, and 0.93, respectively). The gas production increased by increasing the level of Spirulina. However negative linear relationships were recorded between gas production per g DM, OM, DDM and alfalfa substitution level (R^2^ = 0.97, 0.95, and 0.96, respectively) which the gas production decreased as the substitution level increased. In vitro degradability of dry and organic matter was decreased by the increment of *Panicum maximum* and Spirulina supplementation levels and vice versa, until 2 mg/g of Spirulina (*p* < .0001). The addition of Spirulina significantly (*p* < .0001) increased total volatile fatty acids (TVFA) and NH_3_ concentration, until 2mg/g, while the addition of *Panicum maximum* hay significantly increased NH_3_ concentration, until it reached at (R4). In conclusion, the substitution of alfalfa hay with graded levels of *Panicum maximum* hay may be reduce nutrients degradability and gas production while supplementing diets with graded level of spirulina improve degradability and ruminal fermentation parameters.

## Introduction

Water scarcity and soil salinity are major constraints for forage crop production in Egyptian deserts. Cultivating plants adapted to these conditions can increase the potential yield of high-quality forage crops (Elbeih 2021). Desert areas in Egypt suffer from a shortage of fodder, especially in the summer, because of water shortage and soil salinity (Mohamed et al. 2007). Feed shortage remains one of the main constraints to developing animal production in Africa. A solution to improve livestock feeding, and productivity, could be the cultivation of forages that can tolerate salinity and water shortages (Adjolohoun et al. 2013). *Panicum maximum* presents one of the greatest potential productions of dry matter in subtropical and tropical environments (Adjolohoun et al. 2013). The use of *Panicum maximum* is a possible alternative for use as a source of energy, due to its high yield as well as seed propagation (Jank et al. 2013). *Panicum maximum*, which is like other tropical grasses, showed a rapid decline in the crude protein with age (Aganga and Tshwenyane 2004), quality can be improved by mixing it with other rich legumes (Adogla-Bessa et al. 2022). Alfalfa (*Medicago sativa L*.) is a perennial legume widely cultivated to provide high-quality forage in the form of hay, silage, and to a lesser extent as a grazing crop, but Alfalfa has higher water requirement compared to other crops (Li et al. 2023). So, intercropping *Panicum maximum* with forage legumes improves the quality of the fodder (Alasa et al. 2014), reduces water consumption, and reduces the feed cost for small breeders, particularly in desert areas. Replacing alfalfa hay with increasing amounts of *Panicum maximum* hay may cause a decrease in the mixture’s nutritive value. However, supplementing the diets with spirulina as a digestive enhancer can improve efficiency of feed utilization. Algae are gaining interest as alternative sources of micronutrients due to their rich functional metabolite content including polysaccharides, proteins, peptides, amino acids, lipids, polyphenols, and minerals (Brown et al. 2014). Han and McCormick (2014) suggested that most algae are considered high protein supplements with high-fat content, soluble carbohydrates, macro and micro minerals, and polyunsaturated fatty acids (PUFA) while having low fiber content. Thus, they are suitable for use as feed additives with health-improving characteristics for livestock. Also, this supplement contains pigments, antioxidants, provitamins, vitamins, growth factors, and all the essential nutrients. The mode of action of algae is based on its content of nutrients, polysaccharides, antioxidants, unsaturated fatty acids, and minerals. These elements work to activate the microflora and modulate gut microbiota composition in the rumen, as confirmed by (Gotteland et al. 2020; Rabee et al. 2022). However, there is limited research on the use of *Spirulina platensis* as a feed additive in ruminant feeding. The present in vitro experiment aimed to evaluate the effect of substituting alfalfa hay with graded levels *panicum maximum* without or with graded levels of spirulina supplementation on rumen fermentation and nutrient degradability on in vitro gas production, nutrient degradability and fermentation parameters.

## Materials and methods

This research was conducted in the Animal and Poultry Nutrition Department labs, Desert Research Center (N 30°07′18.0372", E 31°18′54.3276") and Animal Nutrition Research lab, Animal Production Department Faculty of Agriculture (N 30°06′48.9924", E 31°14′46.2768"), Ain Shams University.

### Forage materials, spirulina supplementation and treatment diets

Alfalfa hay (*Medicago sativa*) was harvested before flowering, and 1 m height from Maryout Research Station (N 30°09′07.5024", E 31°14′28.9824").

*Panicum maximum cv. Mombasa*, was harvested before it could flower and had also grown to a height of 1 m from Siwa Research Station farm (N 29°12′11.4156", E 25°31′10.362"). Both stations are affiliated to the Desert Research Center (DRC), Ministry of Agriculture and Land Reclamation, Cairo, Egypt. The collected plants were sun-dried (temperature 28°C and humidity 33%) for six days until hays were obtained.

Alfalfa hay (*Medicago sativa*) and *Panicum maximum* hays were finely crushed and preserved in plastic bags to prevent the absorption of moisture for further proximate analysis and in vitro experiments. Each fodder sample (500g) was manually chopped using a machete into particles (2–5 cm) and dried at 60oC until constant weight in a ventilated drying oven (Qallenhamk OVE25010G). After drying, samples were crushed using a hammer mill to pass through sieve size 1 mm, and then preserved in plastic sachets. Spirulina extract with 100% purity in powder form was purchased from the Algal Biotechnology Unit in Dokki, Giza, Egypt.

Treatments diets were formulated to be isonitrogenous and isocaloric and contained 40% forage. Forage composition was altered by substituting alfalfa hay with graded levels of *panicum maximum* hay, so that treatment diets contained 0, 25, 50, 75 and 100% of the forage from *panicum maximum* for treatments R1, R2, R3, R4, and R5, respectively. Additionally, each treatment was further supplemented with graded levels of spirulina added at the following rates: 0, 0.5, 1.5, 2, 2.5, and 3 mg/g.

### Analytical methods

Proximate chemical analyses were conducted on the experimental samples for crude protein (CP), crude fiber (CF), ether extract (EE), and total Ash as per the procedures laid down by the Association of Official Analytical Chemists AOAC (1990). Neutral detergent fiber (NDF) and acid detergent fiber (ADF) were determined according to sequential procedures of Van Soest et al. (1991) using Ankom^200^ (Ankom Technology Corp., Fairport, NY) filter bag technique. The diets formulation and chemical compositions of the total diet are presented in Table 1.

**Table 1:** Ingredients of the treatment diets, and their chemical composition on DM basis in the in vitro systems

	R_1_	R_2_	R_3_	R_4_	R_5_	Alfalfa	Panicum	Spirulina
Ingredients	Ingredients of the treatment diets kg/100 kg on DM basis							
Alfalfa hay (A), kg/100 kg	40	30	20	10	0	-	-	-
*Panicum maximum* (P), kg/100 kg	0	10	20	30	40	-	-	-
Corn, kg/100 kg	34.8	33.1	31	29.3	27.5	-	-	-
Soya meal, kg/100 kg	4.5	6.2	7.8	9.5	11.2	-	-	-
Wheat bran, kg/100 kg	19.5	19.5	20	20	20.1	-	-	-
Disodium bicarbonate, kg/100 kg	0.15	0.15	0.15	0.15	0.15	-	-	-
Salt, kg/100 kg	0.4	0.4	0.4	0.4	0.4	-	-	-
Limestone, kg/100 kg	0.5	0.5	0.5	0.5	0.5	-	-	-
Premix, kg/100 kg	0.15	0.15	0.15	0.15	0.15	-	-	-
Chemical composition of the experimental dietary formulations used in the in vitro systems (g/Kg DM basis)								
Ash, g/Kg	69	72	76	81	82	113	139	143
Dry matter, g/Kg	884	888	892	897	900	884	922	899
Organic Matter, g/Kg	931	928	924	920	918	887	861	857
Crude Fiber, g/Kg	153	156	158	160	163	333	353	38
Crude Protein, g/Kg	162	160	161	161	161	171	98	472
Ether Extract, g/Kg	26	27	27	29	30	5	11	60
Nitrogen-Free Extract, g/Kg	590	584	579	569	565	378	399	287
Neutral detergent Fiber, g/Kg	332	335	347	350	360	474	546	398
Acid detergent Fiber, g/Kg	150	156	160	161	164	311	352	189
Non-Fiber Carbohydrate, g/Kg	411	405	389	379	368	237	207	-
Calculated energy contents								
Gross Energy, kcal/ kg DM	3859.74	3854.61	3836.56	3830.82	3830.07	-	-	-
Digestible Energy, kcal/ kg DM	2930	2930	2920	2910	2910	-	-	-
Metabolizable Energy, kcal/ kg DM	2410	2400	2390	2390	2390	-	-	-
TDN, %	66.53	66.44	66.13	66.03	66.02	-	-	-

R_1_: A (100%) + CFM_1_, R_2_: (A75% + P25%) + CFM_2_, R_3_:(A50% + P 50%) + CFM_3_, R_4_:(A25% + P 75%) + CFM_4_, R_5_: P (100) + CFM_5_. DE, ME, GE are calculated according to NRC (2001)

Fatty acids analysis was determined using Gas- Liquid Chromatography (GLC). where the extracted fatty acids of each plant and the standards were converted to the corresponding methyl esters using an ethereal solution of diazomethane (Farag et al. 1980). The methyl esters of the fatty acids were analyzed with Agilent – 8890 gas – chromatographic apparatus. The fraction of fatty acids methyl esters was conducted using GLC column. Peak identification was performed by comparing the relative retention time of each compound with those of standard materials. The relative proportions of each compound were estimated as the ratio of the partial areas to the total area as mentioned by Fryer et al. (1960), Nelson et al. (1969), and Farag et al. (1980). Fatty acid profile for different diet ingredients are presented in Table 2.

**Table 2:** Fatty acid profile in the experimental ingredients used in the in vitro systems

Fatty acid	Alfalfa hay	*Panicum maximum* hay	Spirulina
Acetic	7.92	26.98	2.93
Butric	-	-	2.05
Enanthic	2.56	7.59	2.23
Heneicosanoic	0.87	0.56	6.27
Erucic	0.47	0.53	0.55
Arachidic	0.49	0.52	-
Behenic	0.49	-	-
Caproic	-	-	1.00
Caprylic	-	-	0.64
Capric	-	-	0.74
Undecanoic	-	-	0.73
Lauric	-	-	0.40
Myristic	-	-	1.38
Palmetic	-	-	1.15
Stearic	-	-	0.40
Arachidonic	-	-	2.80
Oliec	-	-	2.04
Linoleic	-	-	1.07
cis-11,14,17-Eicosatrienoic	0.88	0.99	0.91
cis-11,14-Eicosadienoic	0.89	-	7.60
cis-8,11,14-Eicosatrienoic	0.49	-	1.22
cis-5,8,11,14,17- Eicosapentaenoic	16.15	9.95	2.98
cis-13,16-Docosadienoic	0.52	0.48	0.95
cis-11-Eicosenoic acid	0.67	1.23	-
cis-4,7,10,13,16,19-Hexaenoic	0.55	0.89	-
cis-10-heptadecanoic	-	-	1.64

Qualitative phytochemical screening was conducted on the alcoholic extracts of alfalfa hay, *Panicum maximum* hay, and Spirulina extract. The presence of total tannins, saponins, and total phenols was determined using methods described by Makkar et al. (1993), Tava and Avato (2006) (Table 3). Quantitative estimation for total tannins was carried out by gravimetric method (Makkar et al. 1993), saponins and total phenols (Tava and Avato 2006) (Table 3).

**Table 3:** Preliminary phytochemical screening and quantitative estimation of the anti-nutritional factors (ANF’S) in the experimental ingredients

Type	Total tannins mg%	Saponins g/100gDM	Total phenol %
Alfalfa hay	+ +	+ +	+ +
*Panicum maximum* hay	+ + +	ND	+ + +
Spirulina	+	+ + +	+
Concentrations of the anti-nutritional factors (ANF’S) in the experimental ingredients			
Alfalfa hay	2.58	2.64	0.20
*Panicum maximum* hay	3.75	ND	0.25
Spirulina	1.09	3.66	0.17

*ND* Not Detected

^+^present

### In vitro gas production experiment

An in vitro batch culture technique was applied as described by Szumacher-Strabel et al. (2002). The experiment was carried out in triplicates for each treatment. About 500 ± 3 mg of feed (according to the experimental design) was weighed into 120 ml incubation vessels using an electric balance (KERN 770). At least 3 blanks were included, and Alfalfa hay (H), and Concentrate (CONC) were used in triplicate as the standard.

Rumen fluid was obtained from adult sheep aged 2 years, fed clover hay for a two-week immediately after slaughter et al.-Marg slaughterhouse. The collected rumen fluid was mixed and squeezed through a 4-layer cheesecloth into a bottle (2L) with an O2-free headspace and maintained in an insulated container containing warm water 39°c, then immediately transported to the laboratory (Sarkwa et al. 2023).

Buffer solution was prepared according to McDougall the buffer was made up of 9.8 g NaHCO_3_, 2.44 g Na_2_HPO_4_, 0.57 g KCl, 0.47 g NaCl, 0.12 g MgSO_4_.7H_2_O, and 0.16 g CaCl_2_.2H_2_O per liter of distilled water. It is important to note that CaCL_2_ must be added only after all the other components have completely dissolved. During the warming and reducing step, urea is added to the buffer at a rate of 1.0 gm/liter. Rumen fluid was mixed with buffer solution in a ratio of 1:4 (v/v) to use as a source of inoculum. Each vessel was filled with 50 ml of the incubation medium and dispensed anaerobically before being closed. The samples were then incubated at 39°C for 24 h. Finally, the vessels were randomly distributed in the rack in the incubator and the tubes were swirled continuously.

Volumes of gas produced were measured after 24 h using a 100 ml glass syringe as described by Menke and Steingass (1988) and validated by Sarkwa et al. (2020). To calculate the accurate volume of gas produced, the following formula was used: GP (ml/sample) = V_24_—GP_0_ x (F_H_ + F_CONC_)/2, where V_24_ represents the volume of gas produced after 24 h of incubation, and GP_0_ represents the volume of gas produced by the blank after 24 h of incubation. F_H_ represents the volume of gas produced by standard hay/ recorded gas production of standard hay and F_CONC_ represents the volume of gas produced by standard concentrate / recorded gas production of standard concentrate.

After 24 h of incubation, the gas production were recorded. Then, the filtration process was performed on each of the 120 ml vessels using a filter bag (F57 Ankom). The pH values were recorded, and Ammonia and total volatile fatty acids (TVFs) concentrations were determined in the liquid part. After the filtration process the filter bags were dried at 105° C for 3 h in an oven (Qallenhamk OVE25010G) to estimate residual DM, NDF and ADF. The Dry matter Degradability (DMD), Neutral Detergent Fiber Degradability (NDFD) and Acid Detergent Fiber Degradability (ADFD) were calculated as the difference between the weight of the incubated substrate and the weight of non-degraded residue at the end of incubation, according to the following formula (Van Soest et al. 1991):

$$\text{IVD }({\%})=((\text{R}-\text{P}))/\text{R}\times 100$$

where R = weight of the sample inside. P = The true weight of the out sample.

The pH of rumen liquor was immediately recorded using pH meter (Gallen Kamp pH Stick pH K-120 – B). Rumen liquor samples were analyzed to determine ammonia concentration, (NH_3_) by Nessler’s method modified by Szumacher- Strabel et al*.* (2002) and total volatile fatty acids (TVF’s) by steam distillation according to Warner (1964).

After 24 h incubation, the gases produced and corrected by gases of the blank tubes were used to calculate the:

1. In vitro digestibility of organic matter, using the following regression equation (Lemoufouet et al. 2019): OMD (%) = 14.88 + 0.889 GP + 0.45CP + 0.065 Ash.
2. The content of the Metabolizable energy (ME) was calculated according to the following equation (Lemoufouet et al. 2019): ME (MJ/kg DM) = 2.20 + 0.136GP + 0.057CP.
3. Short chain fatty acids (SCFA) were calculated as described by Getachew et al. (2002): SCFA = (0.0222GP)−0.00425.

where, GP = quantity of gas produced for 200 mg DM of sample after 24 hours of incubation, CP = crude proteins.

## Statistical analyses

The experiment was designed as a factorial experiment (5 treatment diets X 7 spirulina supplementation level) in a completely randomized design. Data were statistically analyzed using the statistical analysis system SAS software (SAS 2011). Separation among means was carried out according to Duncan’s multiple-range test (Duncan 1955) when the main factor was significant and at a confidence level of 95%. The collected data were subjected to the analysis of variance in two ways with the interaction, analysis model according to the General Linear Model.

The statistical model was as follows:

$${\text{Y}}_{\text{ijk}}=\mu +\alpha \text{ I}+\beta \text{j}+(\alpha \beta )\text{ I J}+{\text{E}}_{\text{ijk}}$$

where, Y _ijk_ = The K ^th^ Observation on the diet subjected to factors I and J; μ = general average; α I = effect of the type of Hay (i = 1,2,…0.5) I; βj = effect of the Algae (j = 1,2,…,7); e_ij_ = residual error on the ruminal liquid subjected to factors I and J; (α β) I J = effect of the interaction between factors I and J.

## Results

### Nutrient degradability and gas production after 24 h of incubation

**Fig. 1:**
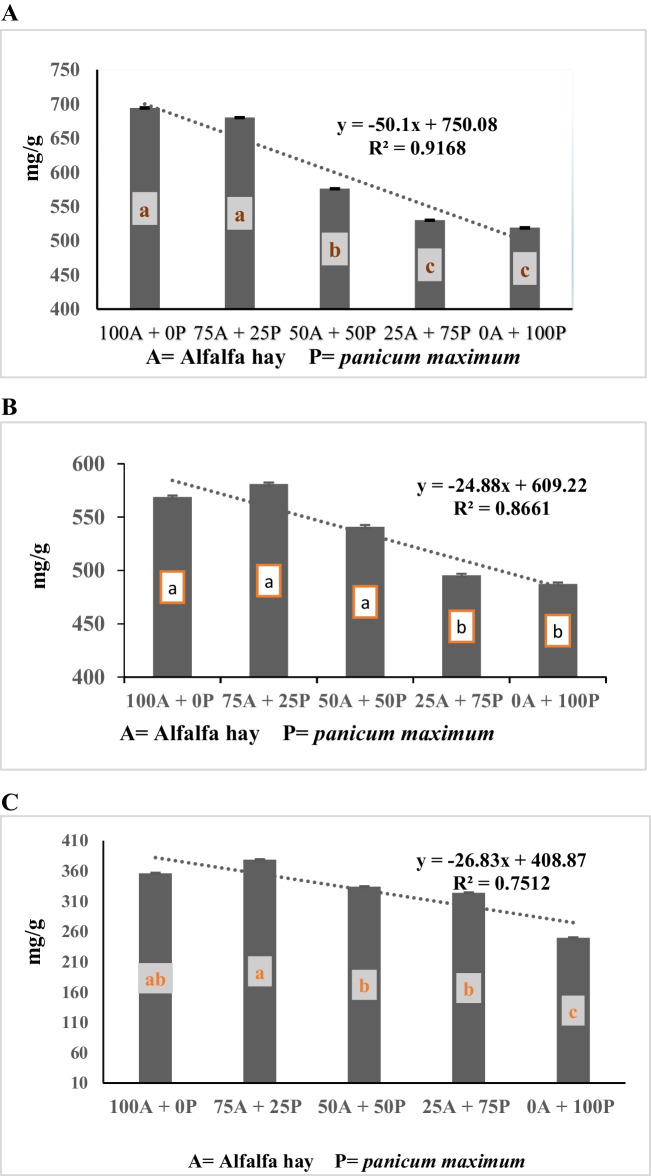
**A** Effect of substituting alfalfa hay with *panicum maximum* hay on in vitro DMD (mg/g). **B** Effect of substituting alfalfa hay with *panicum maximum* hay on in vitro NDFD (mg/g). **C** Effect of substituting alfalfa hay with *panicum maximum* hay on in vitro ADFD (mg/g)

**Fig. 2:**
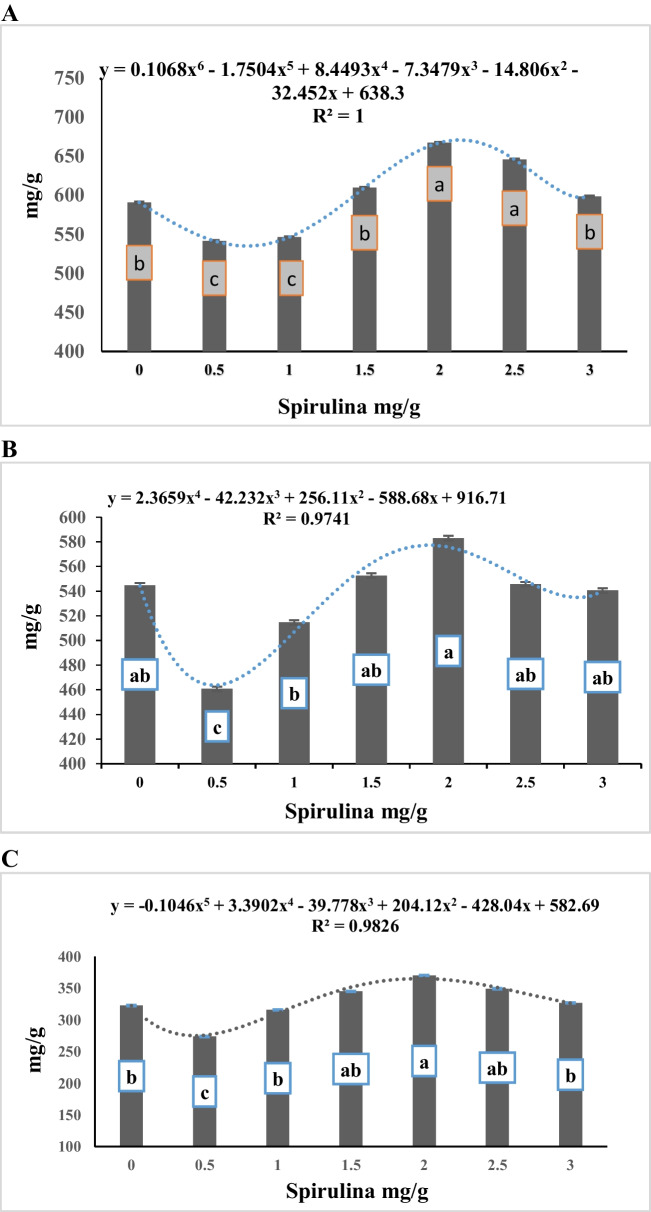
**A** Effect of spirulina addition in vitro DMD (mg/g). **B** Effect of spirulina addition on in vitro NDFD (mg/g). **C** Effect of spirulina addition on in vitro ADFD (mg/g)

The data of Table 4 showed that the interaction between alfalfa hay substitution and spirulina supplementation level was not significant (*P* > 0.05) for degradation of DM, NDF and ADF. However, the data presented in Table 4 and Fig. (1A, B, C) show a negative linear relationship between alfalfa hay substituting level with *panicum maximum* and degradation of DM, NDF, and ADF (R^2^ = 0.92, 0.87, and 0.75, respectively). The data showed a significant (*p* < 0.0001) gradual decrease in DMD with the ascending substitution of alfalfa hay with *panicum maximum* hay, with no significant difference between the control ratio (100% alfalfa hay, R1) and the diet containing 75% alfalfa hay + 25% *panicum maximum* hay (R2), and both treatments were higher than the values for other substituting levels (50%, 75%, and 100% for R3, R4, and R5, respectively). Also, the diet containing 50% alfalfa hay + 50% *panicum maximum* hay (R3) recorded higher in vitro DM, NDF, and ADF degradability than the diet containing 100% *panicum maximum* (R5). Concerning the effect of spirulina supplementation levels, the data presented in Table 4 and Fig. (2A, B, C) shows polynomial relationship between spirulina supplementation level and degradation of DM, NDF and ADF (R^2^ = 1.0, 0.97, and 0.98, respectively). The data demonstrate that the degradation of DM, NDF, and ADF increases gradually as the concentration of spirulina supplementation increases from 0.5 mg/g to 2.0 mg/g. However, the degradation of DM, NDF, and ADF remain unchanged at 3 mg/g, showing similar values as the control (un-supplemented) group.

**Table 4:** Effect of substituting alfalfa hay with *panicum maximum* with or without using Algae (Spirulina) on in vitro degradability parameters (DMD, NDFD and ADFD) and gas production (GP/DM, GP/OM and GP/DMD)

Forage contents	Spirulina level, mg/g	DMD, mg/g	NDFD, mg/g	ADFD, mg/g	GP, ml/ DM g	GP, ml/ OM g	GP, ml/ DMD g
100% alfalfa hay (R1)	0.0 mg/g	667.9	523.2	333.4	123.95	133.32	156.20
	0.5 mg/g	626	488.8	269.2	133.75	139.88	175.16
	1.0 mg/g	672.2	543.68	324.6	125.28	134.74	224.07
	1.5 mg/g	722.4	569.9	383.6	144.56	145.53	273.65
	2.0 mg/g	754.9	622.78	436.5	134.85	145.07	283.20
	2.5 mg/g	729.5	680.32	357.5	142.14	152.84	283.86
	3.0 mg/g	687.1	544.4	387.8	150.58	161.99	284.96
75% alfalfa hay + 25% Panicum (R2)	0.0 mg/g	660.7	587.6	343.7	123.10	132.95	193.50
	0.5 mg/g	644.4	525.2	310.3	127.43	137.62	201.86
	1.0 mg/g	598.9	568.6	390.2	121.29	130.94	199.71
	1.5 mg/g	686.1	629	393.7	131.08	141.54	212.29
	2.0 mg/g	768.3	588.4	421.2	118.99	128.50	265.46
	2.5 mg/g	722.4	607.2	396.9	137.83	148.84	265.86
	3.0 mg/g	679.9	546.3	382.2	148.66	160.57	266.11
50% alfalfa hay + 50% Panicum(R3)	0.0 mg/g	613.5	561.2	322.4	120.72	120.92	174.62
	0.5 mg/g	480.3	424.9	282.8	119.74	128.87	218.07
	1.0 mg/g	505.1	534.4	313.3	128.23	139.05	169.34
	1.5 mg/g	574.8	569.5	349.1	117.47	127.41	216.52
	2.0 mg/g	637.7	600.8	389.4	132.14	143.34	261.07
	2.5 mg/g	618.1	526.9	354.7	126.30	136.96	262.02
	3.0 mg/g	601.5	553.8	312.3	143.71	155.88	261.93
25% alfalfa hay + 75% Panicum(R4)	0.0 mg/g	500.2	525.3	315.2	111.59	121.67	159.24
	0.5 mg/g	488.6	423.9	291	113.48	123.73	170.13
	1.0 mg/g	463.4	468.5	314.5	112.46	122.61	218.60
	1.5 mg/g	509.9	505.9	336.6	122.39	133.46	235.79
	2.0 mg/g	618	550.2	352.4	132.79	144.78	236.75
	2.5 mg/g	606.7	469.1	337.4	115.72	127.29	237.11
	3.0 mg/g	522.4	523.7	320.1	141.62	154.44	237.16
100% Panicum(R5)	0.0 mg/g	510.3	519.3	291.8	106.48	116.51	194.82
	0.5 mg/g	467.7	443.9	216.3	98.73	107.99	181.19
	1.0 mg/g	491.2	455.4	235.7	107.13	117.20	213.28
	1.5 mg/g	553.6	486.4	243.2	105.67	115.53	168.91
	2.0 mg/g	556.6	549.6	248.1	106.77	116.83	245.03
	2.5 mg/g	551.3	442.1	288.1	123.59	134.93	245.38
	3.0 mg/g	501.3	537.4	227.3	135.30	148.08	245.50
	SE	29.058	38.818	29.252	5.118	3.3739	13.5914
*P* value	Substituting	< .0001	< .0001	< .0001	< .0001	< .0001	0.0010
	Algae	< .0001	0.0008	0.0001	< .0001	< .0001	< .0001
	Interaction	0.6679	0.6848	0.9144	0.0715	< .0001	0.0003

*DMD* Dry matter Degradability, *NDFD* Neutral Detergent Fiber Degradability, *ADFD* Acid Detergent Fiber Degradability, *GP/ DM* Gas production/ Dry matter, *GP/ OM* Gas production/ Organic matter, *GP DMD* Gas production / Dry matter degradability, *SE* Standard error (Interaction)

**Fig. 3:**
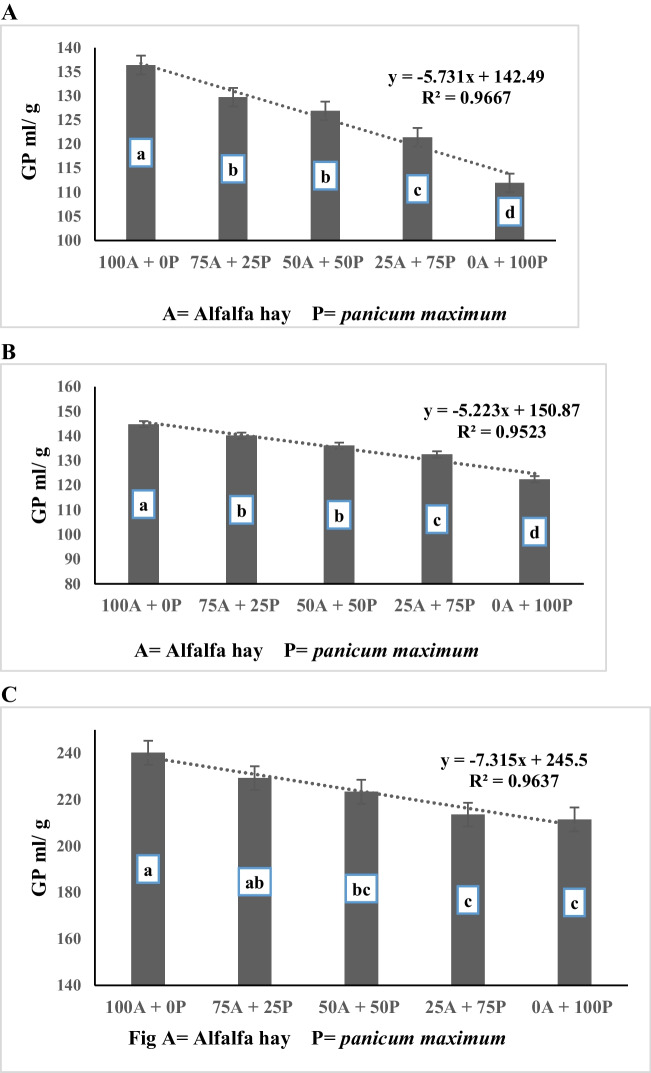
**A** Effect of substituting alfalfa hay with *panicum maximum* hay on in vitro GP/DM (ml/g). **B** Effect of substituting alfalfa hay with *panicum maximum* hay on in vitro GP/OM (ml/g). **C** Effect of substituting alfalfa hay with *panicum maximum* hay on in vitro GP/DMD (ml/g)

**Fig. 4:**
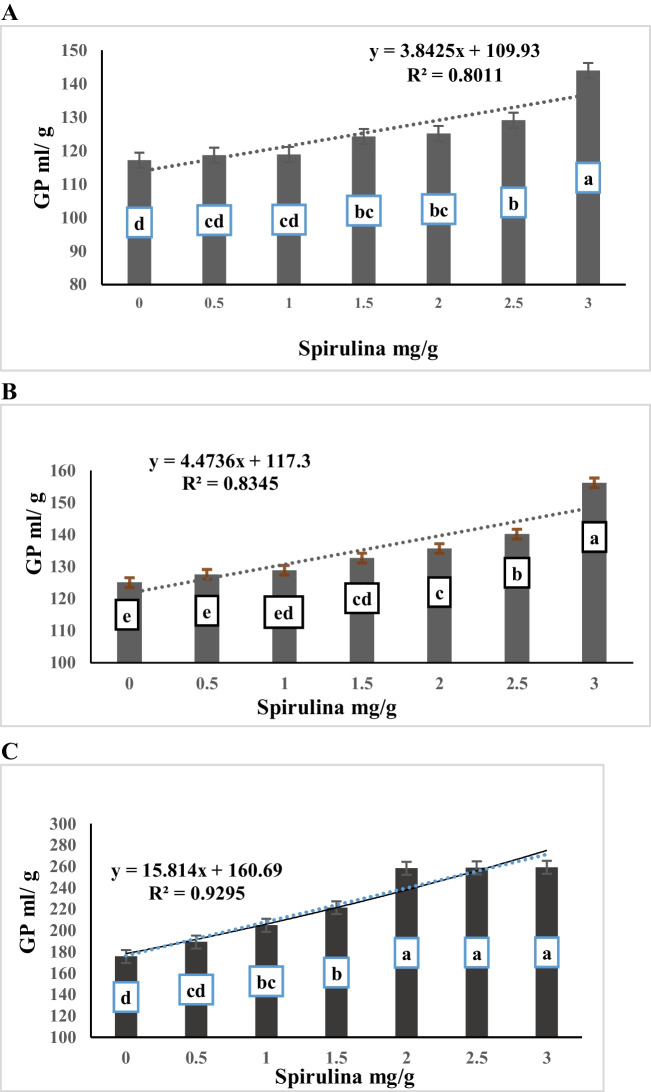
**A** Effect of spirulina addition on in vitro GP/DM (ml/g). **B** Effect of spirulina addition on in vitro GP/OM (ml/g). **C** Effect of spirulina addition on in vitro GP/DMD (ml/g)

The data presented in Table 4 showed a significant interaction (*P* < 0.0001) for gas production per g OM, and g DDM between alfalfa hay substitution and spirulina supplementation level. While insignificant interaction was recorded for (GP per g DM (*P* > 0.05) The treatment diet R1 and supplemented with spirulina at rate of 3 mg/g recorded higher values for gas production (DM, OM, and DMD) followed by the treatment diet R2 and supplemented with 3 mg/g spirulina while the lowest values for gas production were recorded for R5 and supplemented with 0.5 mg/g spirulina. Moreover, the data of Table 4 and Fig. (3A, B, C) show a negative linear relationship between alfalfa hay substituting level with *panicum maximum* and gas production per g DM, OM and DDM (R^2^ = 0.97, 0.95, and 0.96, respectively). The data showed that there is a significant (*p* < 0.0001) gradual decrease in gas production parameters (GP per g DM, g OM, and g DDM) with an increased in the amount of *panicum maximum* hay substituted for alfalfa hay increases. The control ratio (R1), which contained 100% alfalfa hay as forage portion, recorded higher gas production parameters compared to the values for other substituting levels (50%, 75%, and 100% for R3, R4, and R5, respectively). Both diets containing 25% and 50% *panicum maximum* hay as a substitute for alfalfa hay (R2 and R3) were higher than the diet containing 75% and 100% *panicum maximum,* with no significant differences between R2 and R3. Concerning to effect of the spirulina supplementation levels, the results displayed in Table 4 and Fig. (4A, B, C) reveal a positive linear relationship between spirulina supplementation level and gas production per g DM, OM and DDM (R^2^ = 0.80, 0.84, and 0.93, respectively). The data showed a gradual increase (*p* < 0.0001) in gas production parameters (GP per g DM, g OM, and g DDM) as the concentration of spirulina supplementation increases. The data indicates that the diets supplemented with 3 mg spirulina per g of feed had the highest (*p* < 0.0001) gas production per g DM and g OM compared to the other supplemented diets (2.5, 2, 1.5, 1, 0.5, and 0 mg spirulina per g of feed). The diets supplemented with 2.5 and 2 mg spirulina per g of feed also showed higher gas production per g DM and g OM compared to the control diets (not supplemented). However, all the diets supplemented with 3, 2.5, and 2 mg spirulina per g of feed recorded higher gas production per g DMD compared to the diets supplemented with 1.5, 1, 0.5, and zero mg spirulina per g of feed, with no significant differences among them.

### Fermentation and calculated parameters after 24 h

**Fig. 5:**
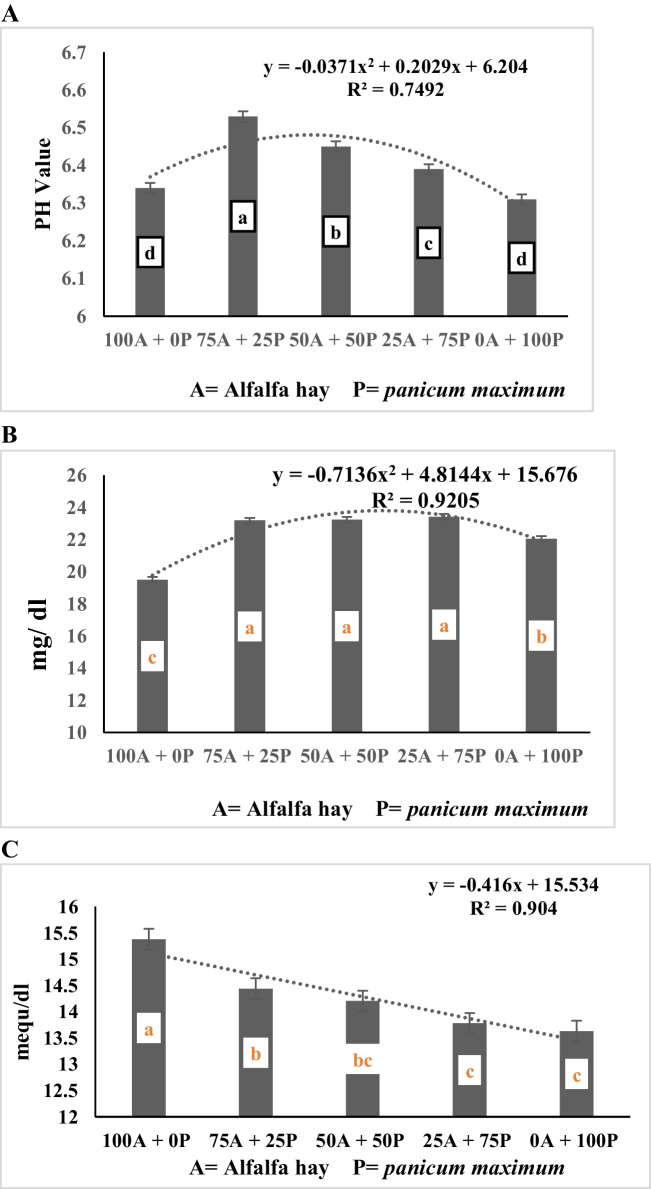
**A** Effect of substituting alfalfa hay with *panicum maximum* hay on in vitro PH. **B** Effect of substituting alfalfa hay with *panicum maximum* hay on in vitro NH3 (mg/ dl). **C** Effect of substituting alfalfa hay with *panicum maximum* hay on in vitro TVFA (mequ/dl)

**Fig. 6:**
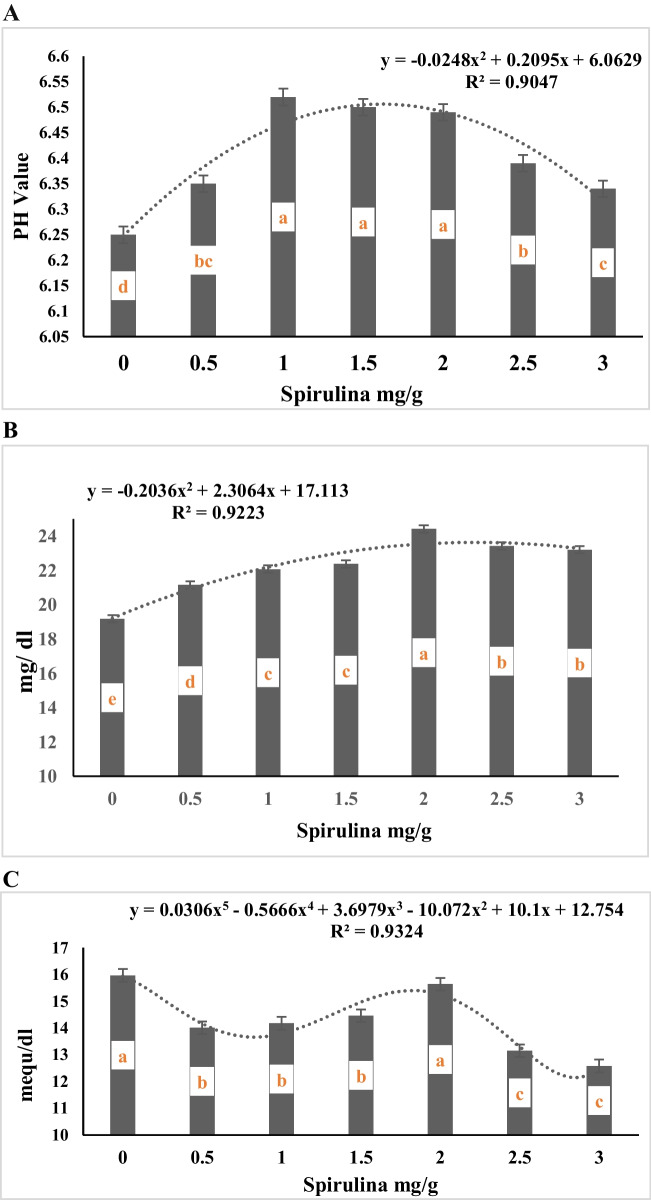
**A** Effect of spirulina addition on in vitro PH. **B** Effect of spirulina addition on in vitro NH3 (mg/ dl). **C** Effect of spirulina addition on in vitro TVFA (mequ/dl)

The data of Table 5 showed that the interaction between substituting alfalfa hay with *panicum maximum* hay and spirulina supplementation level was significant (*p* < 0.0001) on in vitro serum fermentation parameters. The in vitro serum pH values of the different experimental diets were within the normal range, and no abnormal values were observed. The highest pH value was recorded for treatment diets R4 with 1.5 mg/g spirulina followed by the value of R2 with 1 mg/g spirulina supplementation, while the lowest value was recorded for treatment diet R5 and not supplemented with spirulina. The highest ammonia concentrations was recorded for treatment diets R2 with 2 mg/g spirulina supplementation followed by the value of R3 with 2.5 mg/g spirulina supplementation, while the lowest value was recorded for treatment diet R1 and not supplemented with spirulina. The data of Fig. (5A) showed polynomial relationship between alfalfa hay substituting level with panicum maximum and PH values (R^2^ = 0.75), while the data of Fig. (6A) showed polynomial relationship between spirulina supplementation level and PH values (R^2^ = 0.90). The data indicates that increase in pH value as the level of spirulina supplementation increased up to 1 mg spirulina per g of feed. There were no significant differences among spirulina supplementation levels of 1, 1.5 mg/g of feed. The data presented in Fig. (5B and 6B) showed no significant differences among the diets containing 25%, 50%, and 75% *panicum maximum* as a substitute for alfalfa hay in ammonia concentration after 24 h of in vitro fermentation. The lowest significant ammonia concentration was recorded for R1 and not supplemented with spirulina. The data of Fig. (5B) showed polynomial relationship between alfalfa hay substituting level with *panicum maximum* and ammonia concentration (R^2^ = 0.92), while the data of Fig. (6B) showed polynomial relationship between spirulina supplementation level and ammonia concentration (R^2^ = 0.92). The data indicates that gradually increased in ammonia concentration with an increase in spirulina supplementation until 2 mg/g, and then decreased again to a lower concentration at 3 mg/g of feed.

**Table 5:** Effect of substituting alfalfa hay with *panicum maximum* hay with or without using Algae (Spirulina) on in vitro rumen liquor parameter (PH—NH3 – T.VFA) and in vitro calculated parameter (IVDOM, ME and SCFA)

Forage contents	Spirulina level, mg/g	pH value	Ammonia, mg/ dl	Total VFA, mequ/dl	OMD, mg/g	ME, MJ/kg DM	SCHFA, ml/g DM
100% alfalfa hay (R1)	0.0 mg/g	6.16	14.78	14.85	369.5	6.60	0.54
	0.5 mg/g	6.23	17.49	16.22	374.1	6.67	0.55
	1.0 mg/g	6.43	19.65	17.43	388.9	6.81	0.58
	1.5 mg/g	6.33	19.69	12.60	383.4	6.89	0.59
	2.0 mg/g	6.76	21.91	11.95	401.9	7.09	0.62
	2.5 mg/g	6.20	21.31	16.32	406.2	7.16	0.64
	3.0 mg/g	6.26	20.80	15.13	416.9	7.32	0.66
75% alfalfa hay + 25% Panicum (R2)	0.0 mg/g	6.40	21.73	13.49	358.1	6.43	0.51
	0.5 mg/g	6.56	21.88	14.43	368	6.58	0.55
	1.0 mg/g	6.80	22.60	16.13	371.6	6.64	0.54
	1.5 mg/g	6.76	22.92	13.10	375.4	6.69	0.56
	2.0 mg/g	6.36	25.39	12.45	382.2	6.80	0.57
	2.5 mg/g	6.33	23.92	16.80	394.2	6.98	0.61
	3.0 mg/g	6.50	23.81	14.30	413.5	7.28	0.65
50% alfalfa hay + 50% Panicum (R3)	0.0 mg/g	6.23	19.80	13.65	356.3	6.41	0.51
	0.5 mg/g	6.40	22.21	14.00	362	6.49	0.52
	1.0 mg/g	6.53	22.69	16.05	363.8	6.52	0.53
	1.5 mg/g	6.23	23.05	11.86	373.7	6.67	0.55
	2.0 mg/g	6.70	25.27	12.75	374.9	6.69	0.56
	2.5 mg/g	6.66	25.00	14.70	384.1	6.83	0.58
	3.0 mg/g	6.43	24.62	13.19	404.7	7.15	0.63
25% alfalfa hay + 75% Panicum (R4)	0.0 mg/g	6.36	18.47	13.76	347.6	6.29	0.48
	0.5 mg/g	6.33	23.05	14.18	349.1	6.31	0.49
	1.0 mg/g	6.43	23.65	14.60	350.9	6.34	0.49
	1.5 mg/g	6.82	24.12	13.07	354.9	6.40	0.50
	2.0 mg/g	6.36	25.69	12.95	364.8	6.55	0.53
	2.5 mg/g	6.16	24.56	14.85	383.3	6.83	0.57
	3.0 mg/g	6.23	24.37	16.22	400.9	7.10	0.62
100% Panicum (R5)	0.0 mg/g	6.10	20.41	13.16	325.8	5.97	0.43
	0.5 mg/g	6.23	21.15	11.66	337	6.14	0.46
	1.0 mg/g	6.40	21.86	15.15	337.3	6.14	0.46
	1.5 mg/g	6.36	22.12	13.46	339	6.17	0.46
	2.0 mg/g	6.23	23.91	13.96	339.6	6.18	0.47
	2.5 mg/g	6.60	22.41	15.15	367.6	6.60	0.54
	3.0 mg/g	6.26	22.49	12.83	389.7	6.95	0.59
	SE	0.036	0.474	0.5312	9.418	0.1277	0.0234
P value	Substituting	< .0001	< .0001	< .0001	< .0001	< .0001	< .0001
	Algae	< .0001	< .0001	< .0001	< .0001	< .0001	< .0001
	Interaction	< .0001	< .0001	< .0001	0.9998	0.9992	0.9999

*OMD* Organic Matter Digestibility, *ME* Metabolizable Energy, *SCFA* Short Chain Fatty Acid, *SE* Standard error (Interaction)

The data of Fig. (5C) showed a negative linear relationship between alfalfa hay substituting level with *panicum maximum* and TVFA’s concentration (R^2^ = 0.90), the data indicates that a significant gradual decrease in TVFA’s with the ascending substitution of alfalfa hay with *panicum maximum* hay. The control ratio (R1) was higher VFAs concentration than the values for R2, R3, R4, and R5. The data presented in Table 5 and Fig. (5C and 6C) showed no significant differences among the diets R1 without spirulina and R1 with 2 mg/g spirulina in TVFA’s concentration. The lowest significant TVFA’s concentration was recorded for diets R5 with 0.5 mg/g spirulina. The data in Fig. (6C) showed polynomial relationship between spirulina supplementation level and TVFA’s (R^2^ = 0.93), the data indicates that control diet recorded the highest TVFAs concentration compared to all levels of supplementation. However, there was a significant gradual increase from 0.5 kg up to 2 mg/g of feed, followed by a gradual decrease until 3 mg spirulina supplementation per g of feed.

**Fig. 7:**
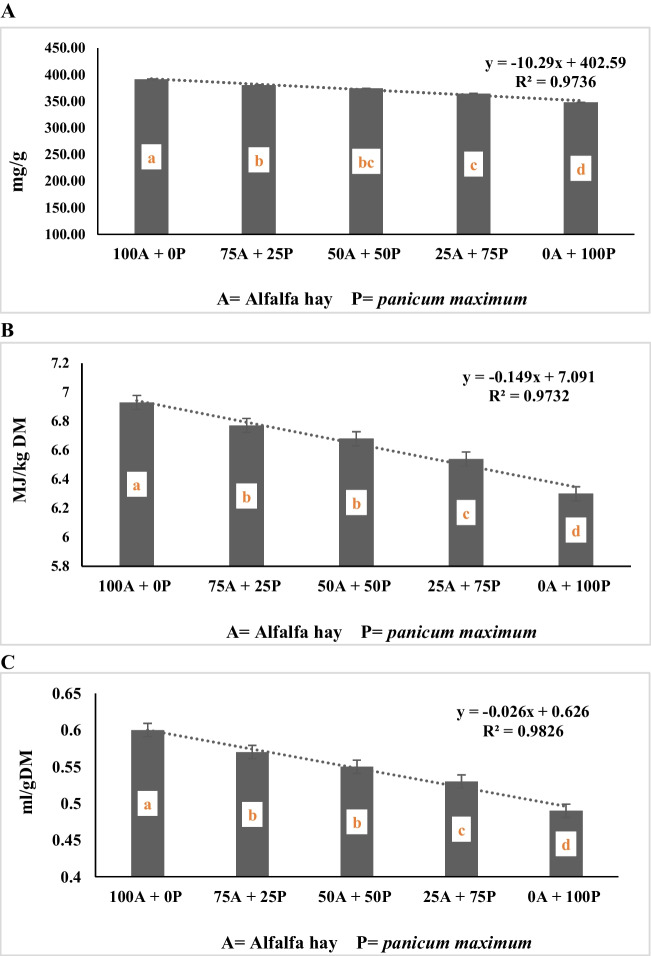
**A** Effect of substituting alfalfa hay with *panicum maximum* hay on in vitro OMD (mg/g). **B** Effect of substituting alfalfa hay with *panicum maximum* hay on in vitro ME (MJ/kg DM). **C** Effect of substituting alfalfa hay with *panicum maximum* hay on in vitro SCFA

**Fig. 8:**
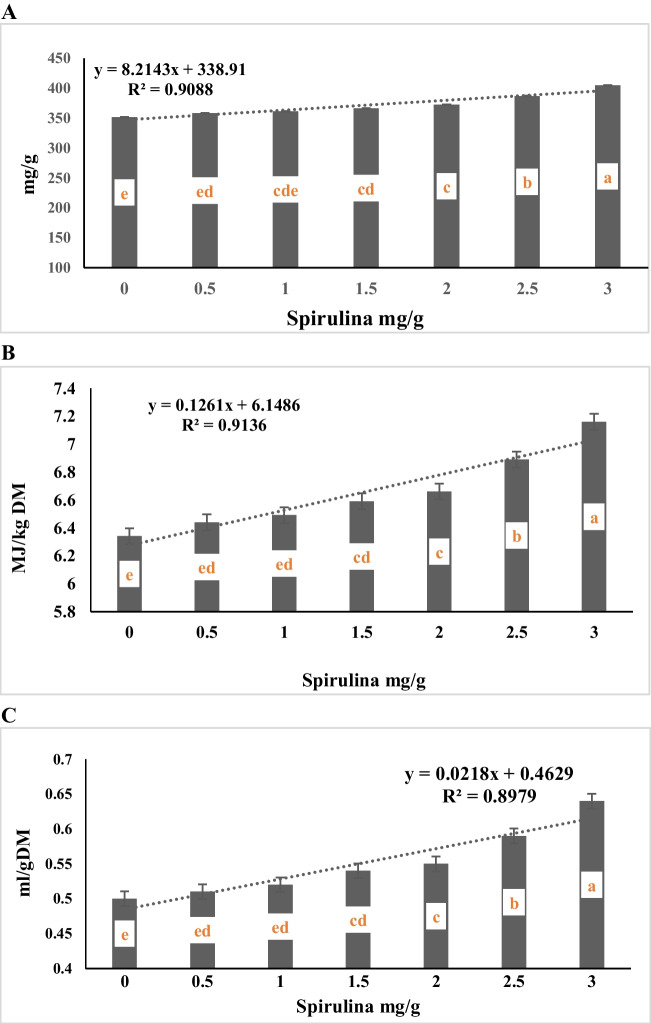
**A** Effect of spirulina addition on in vitro OMD (mg/g). **B** Effect of spirulina addition on in vitro ME (MJ/kg DM). **C** Effect of spirulina addition on in vitro SCFA

The data of Table 4 showed that the interaction between alfalfa hay substitution and spirulina supplementation level was not significant (*P* > 0.05) for organic matter degradability (OMD), ME content and SCHFA. However, the data of Table 5 and Fig. (7A, B, C) show a negative linear relationship between alfalfa hay substituting level with *panicum maximum* and gas production per g OMD, ME and SCHFA (R^2^ = 0.97, 0.97, and 0.98, respectively). The data showed that there is a significant (*p* < 0.0001) gradual decreased in organic matter degradability (OMD) Fig. (7A), ME content Fig. (7B) and SCHFA Fig. (7C) as the amount of *Panicum maximum* hay substituted for alfalfa hay increase. The control ratio (R1), which contains 100% alfalfa hay as forage portion, recorded higher calculated parameters as OMD, ME, and SCHFA compared to the values for other substituting levels (50%, 75%, and 100% for R3, R4, and R5, respectively). Both diets contained 25% and 50% *Panicum maximum* hay as a substitute for alfalfa hay (R2 and R3) were higher in OMD, ME, and SCHFA than the diet containing 75% and 100% *Panicum maximum,* with no significant differences between R2 and R3. Supplementing the experimental diets with ascending levels of spirulina showed a positive linear relationship (R^2^ = 0.91, 0.91, and 0.90, respectively) between the concentration of spirulina and the calculated parameters (OMD, ME and SCFA) until the highest values, which were produced with the highest concentration of spirulina 3 mg/g, Table 5 and Fig. (8A, B, C).

## Discussion

### Nutrient degradability and gas production after 24 h of incubation

The treatment diets were formulated to be isocaloric and isonitrogenous. However, there was a gradual decrease in the degradability of dry matter (DM), neutral detergent fiber (NDF), and acid detergent fiber (ADF) with the increasing substitution of alfalfa hay with *Panicum maximum* hay. This decrease may be attributed to the increase in crude fiber (CF), NDF, ADF, and ash content as the substitution rate increased, along with a decrease in organic matter (OM) and Non fiber carbohydrates (NFC) content. Also, the higher content of tannins and total phenols for *Panicum maximum* hay compared to alfalfa hay. In this connection, Cilliers and Van der Merwe (1993) concluded that the decrease in *Panicum maximum* degradability is associated with a decrease in N content and an increase in NDF, ADF, and ADL contents. Indeed Zhong et al. (2021) reported that dry matter degradation increased in feedstuff with low levels of lignin and NDF because high levels of lignin may resist fiber degrading microorganisms’ activity in the rumen. A similar trend was observed by Ramirez et al. (2009). Also, Yasmin et al. (2008) found that the presence of secondary metabolites or anti-nutritional factors (ANFs) in animal diets affects nutrient digestion and absorption negatively and in this current study *Panicum maximum* contained high levels of tannins (Table 3). Anti-nutritional factors (ANFs) such as tannins, glucosides, flavonoids, alkaloids, terpenoids, cyanides, coumarin, nitrate, oxalate, and organic acids have different effects on animal performance. High levels of ANFs in an animal’s diet prevent the growth of microbes and fungi in the rumen, thus affecting the rate of nutrient digestion and absorption (Acamovic and Brooker 2005). The higher gas production parameters observed in the control diet may be due to the fact that alfalfa is a legume that contains higher CP% and Non fiber carbohydrates (NFC) and lower contents of NDF, ADF, and Ash compared to *Panicum maximum* hay. Concerning the effect of the spirulina supplementation levels on increased degradability, this may be due to several factors, including the high nutrient density of Spirulina and the stimulation of extracellular enzyme secretion by gut microflora (Tovar-Ramírez et al. 2002). Spirulina also contains vitamins, minerals, essential fatty acids, amino acids, and other nutrients that promote faster growth (Costa et al. 2016). Additionally, Spirulina reduces rumen protein degradation and alters bacterial community composition, leading to an increase in the efficiency of rumen microbial crude protein production in steers (Panjaitan et al. 2010). "The increase in gas production with spirulina supplementation levels may be due to the content of nutrients, polysaccharides, antioxidants, unsaturated fatty acids, and minerals in spirulina. These elements work to activate the microflora in the rumen, as confirmed by recent research (Gotteland et al. 2020), spirulina contains all essential amino acids and has a great impact on digestibility (Lafarga et al. 2020; Chia et al. 2019).

### Fermentation and calculated parameters after 24 h

The level of ammonia in rumen liquor is an indicator of nutritional conditions, as many types of rumen micro-organisms use ammonia as a source of nitrogen (Bach et al. 2005). Mixing Alfalfa and *Panicum maximum* showed improved pH and NH_3_ than other treatments containing either of them alone. This may be attributed to complementarity between Alfalfa and *Panicum maximum,* leading to an improvement in the quality of the fodder, as confirmed by Alasa et al. (2014) who concluded that intercropping grasses (*Panicum maximum)* with forage legumes (*lablab purpureus*) improve the quality of the fodder. Additionally, there was an increase in degradability rate and gas production as substituting levels increased. Elevated values of ammonia were associated with an increased percentage of *Panicum maximum* in diets (R2 and R5). This may be attributed to an increased in CP content of the concentrate diet portion through increasing protein sources, especially Soya meal (highly degradable protein source), to compensate for the low CP content of *Panicum maximum* to get iso protein diets. This is in line with Jahan et al. (2018), who reported that many nitrogenous substances in the high concentration diet may be the possible reason for increasing N concentration. Nousiainen et al. (2009) also supported this explanation, where they found that increased concentrate protein feeding improved whole-diet digestibility in cows in a curvilinear manner because of positive effects on the ruminal environment for fiber digestion, but the magnitude of the effect was not very large. Regarding the volatile fatty acids, carbohydrates are fermented by a variety of bacteria in the rumen and transformed into volatile fatty acids (VFA) by the corresponding enzymes (Wang et al. 2020). The data showed a significant decrease in TVFA with the ascending substitution of alfalfa hay with *Panicum maximum* hay. The control ratio (R1) was higher than the values for R2, R3, R4, and R5. This may be due to the low content of fiber fractions (NDF and ADF) and Ash in alfalfa hay compared to *Panicum maximum* hay, with comparable contents of carbohydrates (NFC contents) in both alfalfa and *Panicum maximum* hays. Additionally, the result of an increase in degradability (DM, NDF, and ADF), gas production, and energy (OMD, ME, and SCFA). All these results improve the fermentation process, leading to an increment of VFA. The inclusion of 2 mg/g spirulina resulted in a significant (*P* < 0.0001) increase in pH value and ammonia concentration, without affecting the value of TVFA concentration. These results may be due to the high nutrient density of Spirulina and the stimulation of extracellular enzyme secretion by gut microflora (Tovar-Ramírez et al. 2002). Spirulina also contains vitamins, minerals, essential fatty acids, amino acids, and other nutrients that promote faster growth (Costa et al. 2016). Similar observations were reported by Panjaitan et al. (2010) feeding *Spirulina platensis* as a supplement along with low CP containing guinea grass (*Panicum maximum)* hay improved efficiency of microbial protein production in cattle. Panjaitan et al. (2015) increased microbial protein synthesis and rumen ammonia-N in a quadratic fashion with increasing Spirulina inclusion in the diet. The differences between this study and our findings may be due to the differences in the experimental condition, diet composition and levels of supplementation as well as type of animals.

It’s clear that there is an inverse relationship between the ratio of *Panicum maximum* in diet, and the calculated parameters (OMD, ME and SCFA). This may be attributed to decrease CP and EE contents in diets with panicum hay. This observation was in line with the reported work of Blummel and Orskov (1993) there is a positive correlation between the calculated metabolizable energy from in vitro gas production together with CP and EE contents as well as ME value of conventional feeds measured in vivo. Decrease OM contents and increased of CF, NDF and ADF in diets with Panicum maximum hay, The low OMD and ME obtained for the level of panicum in the treatments increased might probably connected with the presence of high fiber (Blummel and Orskov 1993) especially in treatment R5 (*Panicum maximum* 100%). The relationship between SCFA and gas production was reported by Blummel et al. (1999) who gas production is closely related to SCFA production. Getachew et al. (2002) the SCFA estimated from in vitro* gas* production, has been widely used to evaluate the energy value of several feed classes. Blummel and Orskov (1993) who suggested that gas production from different classes of feeds incubated in vitro in buffered rumen fluid was closely related to the production of SCFA which was based on carbohydrate fermentation. The present findings indicated that all in vitro parameters are affected by the concentration of panicum in diet. Supplementing the experimental diets with ascending levels of spirulina showed a positive relationship between the concentration of spirulina and the calculated parameters (OMD, ME and SCFA), which were produced with the highest concentration of spirulina 3 mg/g. Referring to increased values of calculated ME with increasing spirulina concentration proved that Spirulina specifically 3 mg/g has a good potential to enhance energy content of roughage feedstuffs. Moreover, as the level of SCFA, which is an indicator of the energy content of the diet, its production increased with increasing spirulina concentration in diet. This is due to the fact that Spirulina species, known as cyanobacteria, contain the essential fatty acids, linoleic acid (LA, 18:2 delta-9,12) and gamma-linolenic acid (GLA, 18:3 delta-6,9,12) (Gupta et al. 2008), high quality proteins, carbohydrates, vitamins (B1, B2, tocopherols), minerals (sodium, potassium, calcium, magnesium, phosphorus, iron), carotenes (especially beta-carotene), chlorophyll a, phycocyanin, and some phenolic acids (Gupta et al. 2008). Consequently, diets associated with spirulina makes it possible to raise digestion rates, the energy supply and mineral elements to the microbes present in the rumen fluid, thus improve their growth and activity, and thus, increase the rates of degradability of OMD, ME and SCFA.

## Conclusion

Replacing alfalfa hay with increasing levels of *Panicum maximum* hay resulted in a gradual decrease in the degradability of dry matter (DM), neutral detergent fiber (NDF), and acid detergent fiber (ADF), as well as metabolizable energy (ME) and volatile fatty acid (VFA) concentration. Adding Spirulina as a feed additive (2mg/g) to the experimental diets improved the degradability of nutrients and gas production in the different experimental diets. Further research is necessary to determine the optimal level of substituting alfalfa hay with *Panicum maximum* and the best level of Spirulina supplementation.

## Data Availability

The datasets generated and analyzed during the current study will be provided upon reasonable request from the corresponding author.
